# Development of machine learning models for diagnosis of glaucoma

**DOI:** 10.1371/journal.pone.0177726

**Published:** 2017-05-23

**Authors:** Seong Jae Kim, Kyong Jin Cho, Sejong Oh

**Affiliations:** 1 Department of Ophthalmology, Gyeongsang National University College of Medicine, Jinju, Korea; 2 Department of Ophthalmology, Dankook University College of Medicine, Cheonan, Korea; 3 Department of Software Science, Dankook university, Yongin, Korea; Harbin Institute of Technology Shenzhen Graduate School, CHINA

## Abstract

The study aimed to develop machine learning models that have strong prediction power and interpretability for diagnosis of glaucoma based on retinal nerve fiber layer (RNFL) thickness and visual field (VF). We collected various candidate features from the examination of retinal nerve fiber layer (RNFL) thickness and visual field (VF). We also developed synthesized features from original features. We then selected the best features proper for classification (diagnosis) through feature evaluation. We used 100 cases of data as a test dataset and 399 cases of data as a training and validation dataset. To develop the glaucoma prediction model, we considered four machine learning algorithms: C5.0, random forest (RF), support vector machine (SVM), and *k*-nearest neighbor (KNN). We repeatedly composed a learning model using the training dataset and evaluated it by using the validation dataset. Finally, we got the best learning model that produces the highest validation accuracy. We analyzed quality of the models using several measures. The random forest model shows best performance and C5.0, SVM, and KNN models show similar accuracy. In the random forest model, the classification accuracy is 0.98, sensitivity is 0.983, specificity is 0.975, and AUC is 0.979. The developed prediction models show high accuracy, sensitivity, specificity, and AUC in classifying among glaucoma and healthy eyes. It will be used for predicting glaucoma against unknown examination records. Clinicians may reference the prediction results and be able to make better decisions. We may combine multiple learning models to increase prediction accuracy. The C5.0 model includes decision rules for prediction. It can be used to explain the reasons for specific predictions.

## Introduction

Glaucoma is characterized by dysfunction and loss of retinal ganglion cells (RGCs), with resulting structural changes to the optic nerve head, retinal nerve fiber layer (RNFL) thickness, and ganglion cell and inner plexiform layers as well as loss of the visual field [1].

The diagnosis of glaucoma in its early stages is challenging. Misdiagnosis can lead to failure to identify individuals with the condition during its early stages until significant functional loss has occurred. Thus, early detection of glaucoma allows for early treatment to delay vision loss [2,3]. Diagnosing glaucoma is problematic, especially when it is in the earliest stage of glaucoma. Diagnosis of glaucoma in myopic eyes and patients with brain diseases such as brain tumor is known to be difficult due to those eye’s characteristic disc shape and visual field defect. A more effective glaucoma-detection machine learning model would be very helpful to clinicians.

The classification scheme in machine learning is suitable for diagnosis glaucoma. Chan et al [4] tested various classification algorithms based on the examination of visual fields. Goldbaum et al [5] also compared machine learning classifiers and suggested a mixture of of Gaussian as the best classifier. Bizios et al [6] tested the artificial neural network (ANN) and support vector machine (SVM) based on RNFL thickness parameters. Barella et al [7] investigated the diagnostic accuracy of machine learning classifiers (MLCs) and random forest (RF) using RNFL and optic nerve data. They got 0.877 of area under the ROC value using RF. Recently, Silva et al [8] tested almost all of the classifiers using Spectral Domain optical coherence tomography (OCT) and standard automated perimetry. They got 0.946 as the best aROC value using RF. Previous studies show that SVM and RF have good prediction power. The trade-off between prediction power and interpretability is one of the well-known issues in machine learning. The black box models such as SVM and deep learning algorithm show good prediction power. However, it is difficult to understand why the model gives the prediction result. Therefore, they are not entirely suitable for medical diagnosis because clinicians want to know both the prediction and the reason for the prediction. Decision tree models [9] such as C5.0 [10,11] show good interpretability and poor prediction power. Logistic Regression and Naïve Bayes are algorithms used for probabilistic classification [12]. iDHS-EL [13] and iRSpot-EL [14] are predictors developed for identifying the location of DNase I Hypersensitive Sites (DHSs) and DNA recombination spots in human genomes. The goal of this study is to develop a machine learning model that has strong prediction power for diagnosis of glaucoma. To achieve the goal, we developed good features from examination data for prediction, and we tested C5.0, RF, SVM, and *k*-nearest neighbor (KNN) algorithms. We describe details in the next section.

## Materials and methods

### Procedure

We used three kinds of examination records to develop the learning model: RNFL thickness, visual field (VF) test parameters, and general ophthalmic examination. The records contained glaucoma cases and healthy controls. We extracted as many features (data attributes) as possible from the examination record. All the features were arranged as a data table form except missing values. We performed *t*-tests to evaluate each feature in the data table, and select suitable features for classifying healthy controls and glaucoma.

We divided the base dataset into a test dataset (100 cases) and another dataset (399 cases). Another dataset was used for developing the learning model. We used 80% of it for model training and 20% of it for validation of the model. After finding the best learning model, we evaluated the model using the test dataset.

To develop the learning (glaucoma prediction) model, we considered four machine learning algorithms: C5.0, RF, SVM, and KNN. We repeatedly composed a learning model using training dataset and evaluated it by validation dataset, and a model which showed the best validation accuracy was chosen as the best learning model.

After building the best learning models upon four algorithms, we evaluated the models in various ways. The classification accuracy, sensitivity, specificity, and likelihood ratios were compared. Receiver operating characteristics, (ROC) curves and areas under the curve (AUC) value were also analyzed. In the case of the C5.0 model, this includes a decision tree to predict glaucoma. We analyzed the clinical meanings of the decision rules in the tree. All procedure was implemented by R (http://www.r-project.org) and its supported packages.

### Participants

The medical records of patients who underwent optical coherence tomography (OCT) and VF examinations at Dankook University Hospital and Gyeongsang National University Hospital between January 2012 and November 2015 were reviewed. To conduct the study, all the patients underwent comprehensive ophthalmological examinations, which included slit-lamp biomicroscopy, best corrected visual acuity (BCVAC), refractive error examination, central corneal thickness (CCT) measurement, Goldmann applanation tonometry, dilated fundus examination, and fundus and red-free fundus photography (Canon, Tokyo, Japan). An automated VF test was conducted using the 30–2 program Swedish interactive threshold algorithm standard on a Humphrey 740 visual field analyzer (Carl Zeiss Meditec Inc., Dublin, CA). The spectral-domain OCT (SD OCT) images, obtained using the Spectralis^®^ (Heidelberg Engineering GmbH, Heidelberg, Germany) platform, were used to measure the peripapillary RNFL thickness. This study was approved by the Dankook University Hospital Institutional Review Board, Korea (ID# DKUH 2016-11-011). Informed consent of participants was waived by the Dankook University Hospital Institutional Review Board. This research follows the tenets of the Declaration of Helsinki.

In total, 297 cases of eyes (of patients) with glaucoma (POAG or NTG) and 202 cases of eyes (of patients) without glaucoma were included. The inclusion criteria for glaucomatous eyes were: best-corrected visual acuity of 20/40 or better; normal anterior segment on a slit-lamp examination; and diagnosis of glaucoma by the principal investigator or co-investigator. The glaucoma diagnosis was based on characteristic glaucomatous structural change to the optic disc accompanied by glaucomatous visual field defects. The criteria for a glaucomatous visual field defect were: glaucoma hemifield test [15] outside the normal limit, pattern standard deviation with a P value <5%, or a cluster of <3 points in the pattern deviation plot in a single hemifield (superior or inferior) with a P value of <5%, one of which must have a P value of <1%. Any one of the preceding criteria, if repeatable, was considered sufficient evidence of a glaucomatous visual field defect.

Exclusion criteria were as follows in addition to those who do not met the inclusion criteria: history of ocular inflammation or trauma; and the presence of concurrent retinal disease (i.e., vascular disorder or macular degeneration), optic nerve disease other than glaucoma, or a brain disorder that could influence the visual field results.

The inclusion criteria for normal eyes were a best-corrected visual acuity of 20/40, normal anterior segment on a slit-lamp examination, no RNFL defects in red-free fundus photographs, no visual field defects, and an intraocular pressure ≤21 mmHg. Table 1 summarizes the characteristics of the participants.

**Table 1:** Characteristics of the participants.

	Normal group	Glaucoma group	Total	p-value*
Number of participants	60	110	170	-
Gender (male/female)	32/28	70/40	170	0.2515
Age^¶^ (mean ± SD)	45.8±16.08	61.86±13.91	-	5.086 ×10^−10^
Number of eyes	164	168	332	-
Number of cases	202	297	499	-

SD = standard deviation

*Comparison between glaucoma and normal groups (unpaired *t*-test for Age and chi-square test for Gender).

^¶^ The ages of the participants chosen for the research ranged from 13 years to 90 years, with a mean age of 56.36 years.

### Feature selection and dataset preparation

Table 2 summarizes the basic features that we extracted from the examination records for the glaucoma and healthy controls. To select good features for building the learning model, firstly we removed the features that contained missing values in over 50% of whole cases. We then performed *t*-tests against the rest of the features to see the class separability of the features. As a result, the features 2–8, 10–12 were selected.

**Table 2:** List of basic features from the examination data. We extracted them from examination records for glaucoma and healthy controls.

No	Feature	Source
1	gender	General exam.
2	age	General exam.
3	ocular pressure	General exam.
4	cornea thickness	General exam.
5	RNFL SUP	RNFL
6	RNFL NAS	RNFL
7	RNFL INF	RNFL
8	RNFL TMP	RNFL
9	VFI	VF
10	MD	VF
11	PSD	VF
12	GHT	VF

To increase the quality of the training dataset, we made a synthesized feature. The feature RNFL4.mean reflects the average value of RNFL SUP, INF, and TMP. Because the four RNFL features contain partial information about RNFL, and we need to reflect whole RNFL, we tested every combination of the four RNFL features and SUP-INF-TMP combination showed best performance.

**Fig 1:**
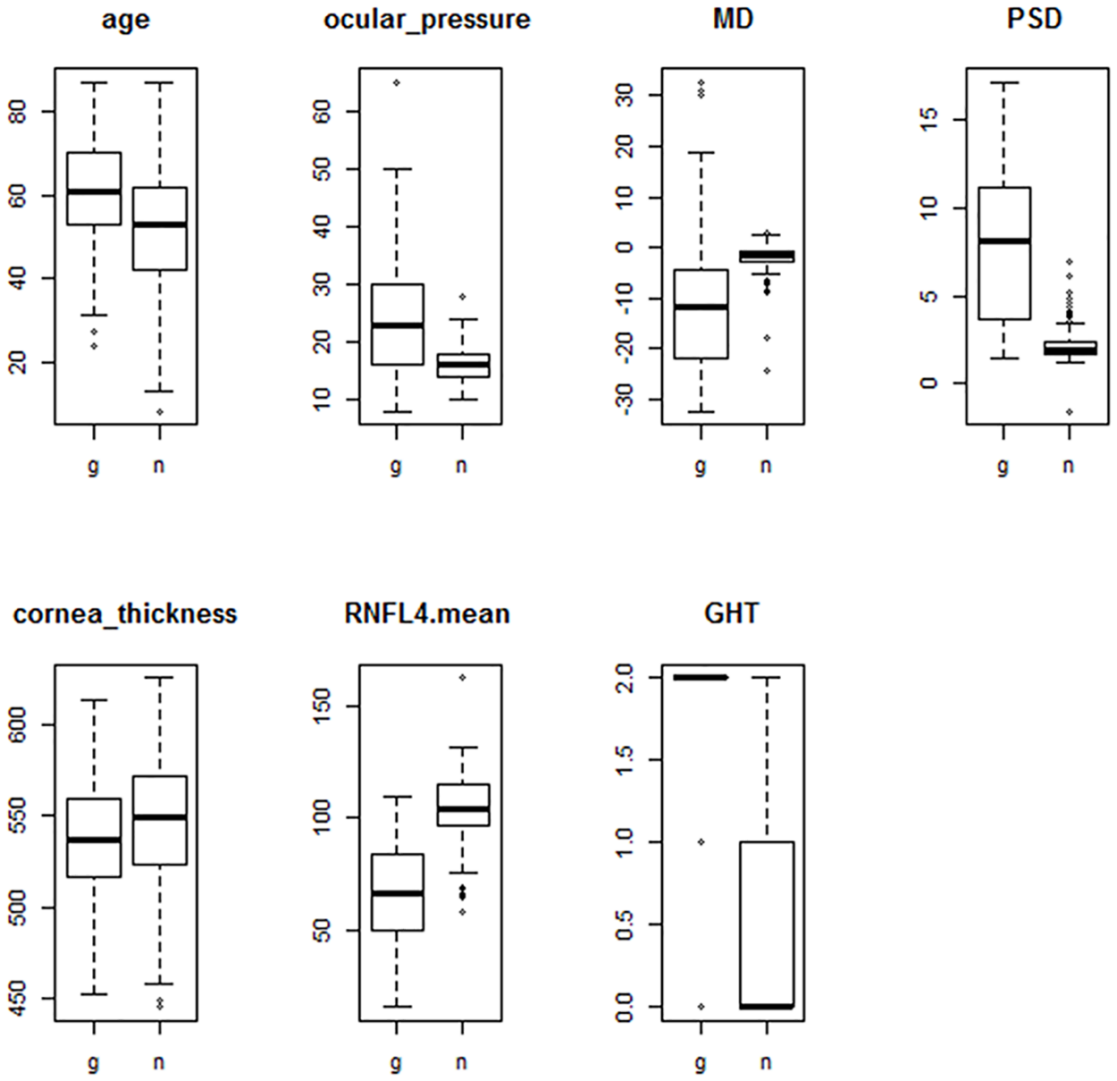
Box plots for selected features (g: Glaucoma, h: Health control). All features show a large difference of median values between glaucoma and healthy controls.

**Fig 2:**
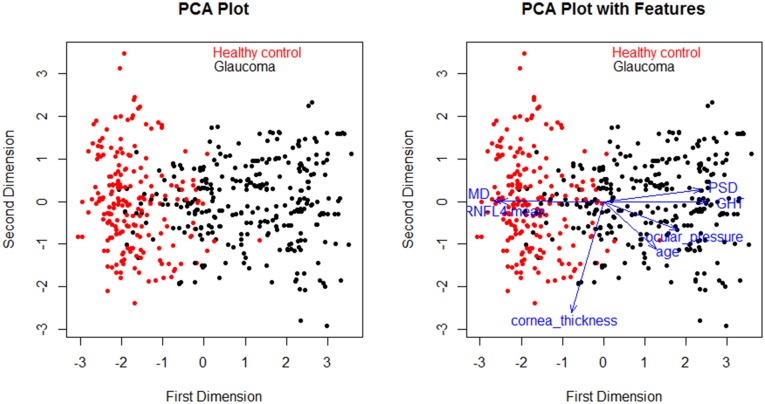
PCA plot for prepared dataset. Each point means a case in the dataset. Generally, the glaucoma cases are well separated from the healthy control cases. Some cases are located in the border area or opposite area. Right plot shows relationship between distribution of cases and features. In the glaucoma group, PSD, GHT, ocular_presure, and age have high values whereas MD and RNFL4_mean have low values.

The final step of feature selection was to sort good features from candidates’ features. We performed a classification test on every combination of the feature subset of candidates’ features using the C5.0 algorithm. Table 3 summarizes the final features from the feature selection process. Fig 1 shows a box plot for the features. All features show a large difference of median value between glaucoma and healthy controls. Fig 2 shows the PCA plot for the prepared dataset. In the plot, each point means a case in the dataset. Generally, the glaucoma cases are well separated from healthy control cases. Some cases are located in border areas or opposite areas. The goal of the learning model may be to correctly predict the cases. The right plot of Fig 2 shows the relationship between distribution of cases and features. In the glaucoma group, PSD, GHT, ocular_pressure, and age have high values whereas MD and RNFL4_mean have low values. In the case of cornea_thickness, the healthy control group has a little bit higher value than glaucoma group.

**Table 3:** Final features list for building the training model. We removed the features that contained many missing values. We then performed *t*-tests against the rest of the features to see class separability of the features. The feature RNFL4.mean reflects mean of SUP-INF-TMP combination.

No	Feature
1	age
2	ocular pressure
3	cornea thickness
4	RNFL4.mean
5	GHT
6	MD
7	PSD

### Learning model evaluation criteria

The *accuracy*, *sensitivity*, *specificity*, *likelihood ratio*, and *ROC*/*AUC* have been widely used as criteria for evaluating a diagnosis model [16]. The following terms are fundamental to understanding the utility of them:

1. True positive (TP): the patient has a disease and the prediction is positive.
2. False positive (FP): the patient does not have a disease but the prediction is positive.
3. True negative (TN): the patient does not have a disease and the prediction is negative
4. False negative (FN): the patient has a disease but the prediction is negative.

The *accuracy* of a diagnosis model refers to the ability of the model to correctly identify those patients with the disease and without the disease:

$$Accuracy=\frac{TP+TN}{TP+FP+TN+FN}$$

The *sensitivity* of a diagnosis model refers to the ability of the model to correctly identify those patients with the disease:

$$Sensitivity=\frac{TP}{TP+FN}$$

The *specificity* of a diagnosis model refers to the ability of the test to correctly identify those patients without the disease:

$$Specificity=\frac{TN}{FP+TN}$$

The *likelihood ratio* is defined as the ratio of expected test results in subjects with a certain disease to the subjects without the disease.^10^ The *Likelihood ratio for positive test results* (*LR+*) tells us how much more likely the positive test result is to occur in subjects with the disease compared to those without the disease:

$$LR+=\frac{Sensitivity}{1-Specificity}$$

The *Likelihood ratio for negative test results* (*LR–*) represents the ratio of the probability that a negative result will occur in subjects with the disease to the probability that the same result will occur in subjects without the disease:^10^

$$LR-=\frac{1-Sensitivity}{Specificity}$$

The *receiver operating characteristic* (*ROC*) plot expresses relationship between sensitivity and 1 –Specificity. The closer the ROC curve is located to upper-left hand corner, the better the model. The *area under the curve* (*AUC*) can have any value between 0 and 1 and it is a good indicator of the goodness of the model.

## Results

### Classification test

**Fig 3:**
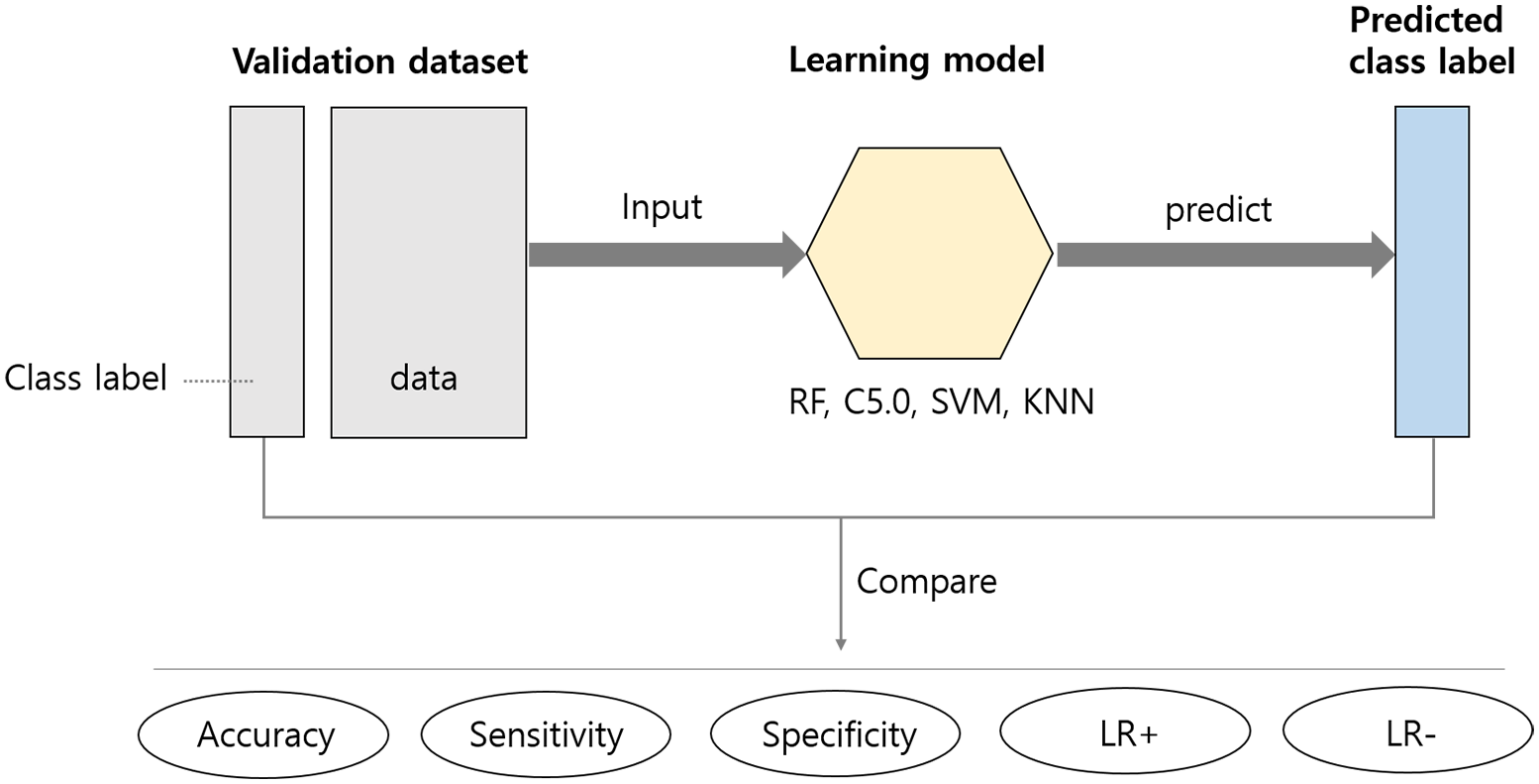
Classification test procedure using learning models.

Fig 3 depicts the classification testing procedure conducted using learning models. From the classification test using the validation dataset, we recorded the statistics as shown in Table 4. As can be seen, the RF model shows the best values on all evaluation criteria. Other models show similar performance.

**Table 4:** Statistics of four learning models from classification tests. The RF model shows the best values on all evaluation criteria. Other models show similar performance.

	Accuracy	Sensitivity	Specificity	LR+	LR-
RF	0.98	0.983	0.975	39.33	0.017
C5.0	0.97	0.983	0.95	19.67	0.018
SVM	0.97	0.983	0.95	19.67	0.018
KNN	0.97	0.967	0.975	38.67	0.034

In *accuracy*, the RF model has a 0.98 rate. The other three models have 0.97. All four models have enough accuracy for medical application.

The *Sensitivity* of RF and C5.0 is 0.983. It means that the proposed three models exactly predict against glaucoma patients and their accuracy is 0.983. It also means that they show a very small FN ratio. In the medical field, FN ratio is more important than FP ratio. Therefore, the thrree models are suitable for diagnosis of glaucoma.

The *Specificity* of the RF and KNN is 0.975. It shows good prediction power against healthy controls. *LR+* is the best indicator for ruling in diagnosis. The higher the *LR+*, the more the test is indicative of a disease. Good diagnostic tests have *LR+* > 10 and their positive result has a significant contribution to the diagnosis [17]. The *LR+* of the RF and KNN models shows 39.33 and 38.67, respectively, and C5.0 and SVM are also larger than 19. *LR*—is a good indicator for ruling out the diagnosis. Good diagnostic tests have *LR–*< 0.1. The lower the *LR–*, the more significant contribution of the test is in ruling out disease [17]. The *LR*—of RF model shows 0.017, C5.0 and SVM shows 0.018, and KNN is 0.034.

Table 5 shows the detailed evaluation results of the RF model. There are two misclassified training samples in the table; one healthy sample is classified into the glaucoma group (FP and one glaucoma sample is classified into the healthy group (FN). In the medical situation, FN is more important than FP. The RF model shows very high accuracy (0.98) and very low FN rate (0.01).

**Table 5:** Classification results of RF model using the test dataset. There are two misclassified training samples in the table, one healthy sample is classified into the glaucoma group (FP and one glaucoma sample is classified into the healthy group (FN).

		Predicted	
		Healthy	Glaucoma
Actual	health (class 0)	39	1
	glaucoma (class 1)	1	59

**Fig 4:**
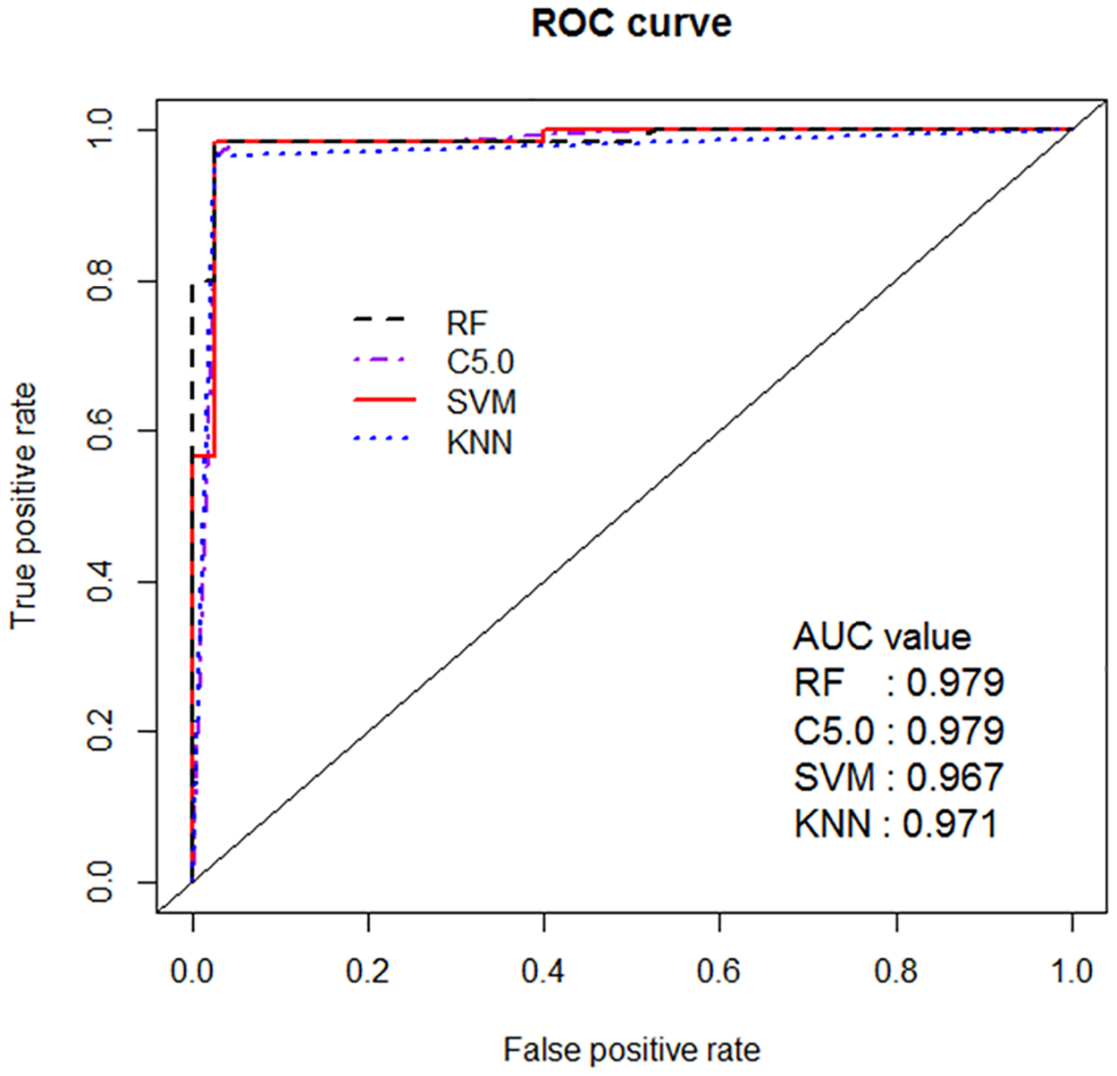
ROC curve and AUC for four models. AUC expresses global quality of prediction models and RF and C5.0 models show 0.979, SVM is over 0.967, and KNN is 0.971. All models show very high values near 1.0.

Fig 4 shows ROC curves and AUC values for all four models. AUC expresses global quality of the prediction model and the RF and C5.0 models show 0.979, SVM is over 0.967, and KNN is 0.971.

Table 6 shows the comparison of model performance between previous works and the proposed RF model. Only AUC of Bizios et al^6^ is higher than the proposed model, but the number of features is much more and sensitivity and specificity are lower than in the proposed model.

**Table 6:** Comparison of previous works and the proposed model. Only AUC of Bizios^6^ is higher than the proposed model, but the number of features is higher and sensitivity and specificity are lower than the proposed model.

Measure	Chan [4]	Goldbaum [5]	Bizios [6]	Barella [7]	Silva [8]	Proposed
ROC	0.923	0.922	0.989	0.877	0.946	0.979
Sensitivity	0.724	0.670	0.968	-	-	0.983
Specificity	0.846	0.790	0.967	0.649	0.951	0.975
# of Features	53	53	17	23	4	7

From the classification test, we reached following conclusions:

1. The quality of our developed features is suitable to use for our glaucoma prediction. It does not depend on any specific learning model. It leads to best evaluation values on the RF model, but it also leads to good evaluation values on C5.0, SVM, and KNN models.
2. The values of measures in Table 4 say that RF, C5.0, and SVM prediction models have very strong and stable potential for glaucoma prediction, with its *sensitivity* measure being very high. Furthermore, the C5.0 model has good interpretability because it is a decision tree model.
3. In conclusion, RF, C5.0, and SVM, based on the proposed features, may be useful for the diagnosis of glaucoma.

### Decision tree of C5.0

**Fig 5:**
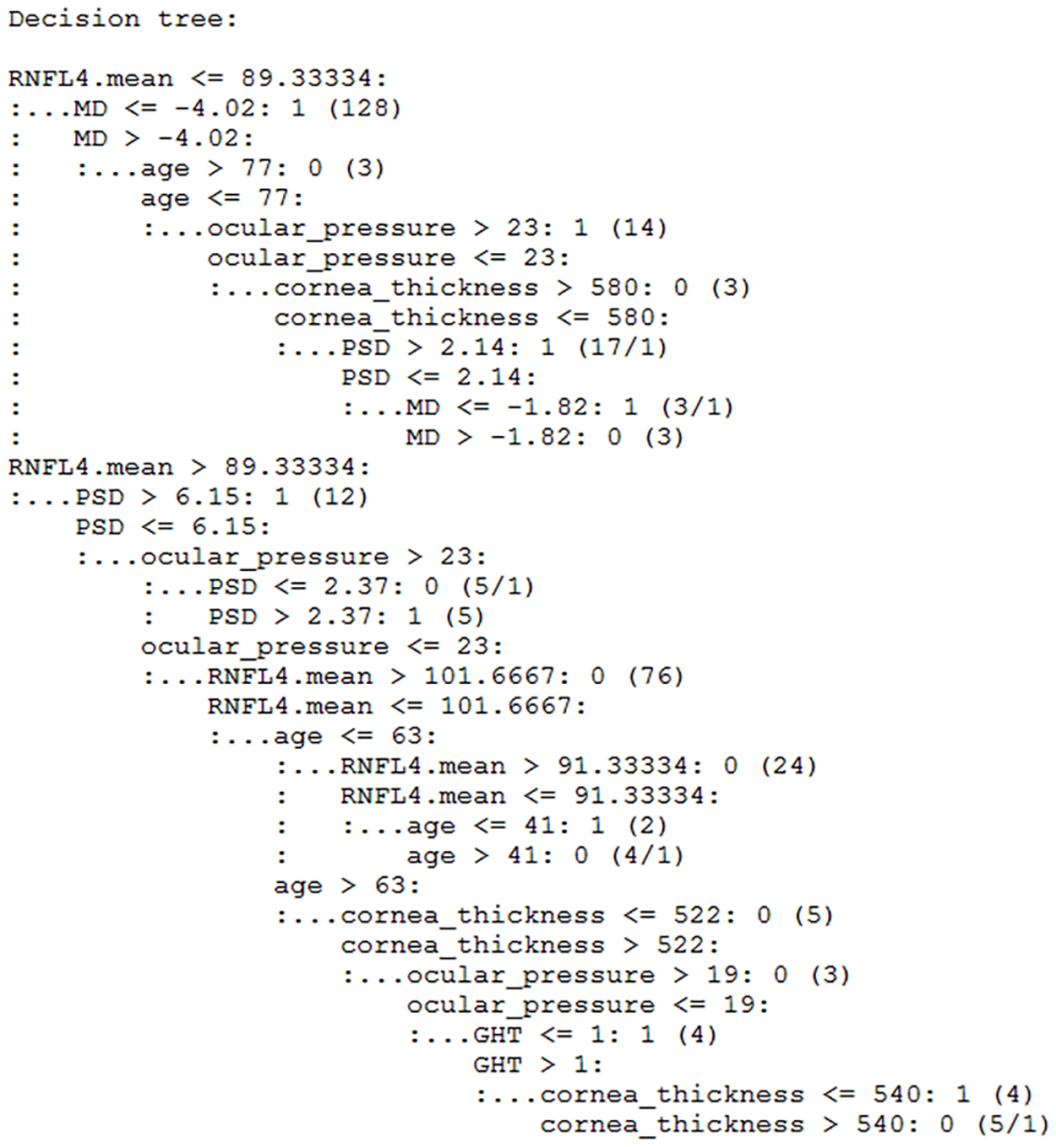
Decision tree for diagnosis of glaucoma from C5.0 algorithm. It contains 19 rules and the training error of the model is 0.016.

C5.0 is an advanced version of ID3 and C4.5 that is developed by Ross Quinlan.^10,11^ C5.0 became an industrial standard for making a decision tree. We used the C50 package in R for testing the C5.0 algorithm. By using the package, we could see and manipulate the structure of decision tree. During the building process of the decision tree, C50 automatically performed the pruning tasks. From the C5.0 model, we constructed a decision tree. Fig 5 shows a whole decision tree. It contains 19 rules and training error of the model is 0.016. Table 7 summarizes usage of features on the decision tree. In C5.0 model, RNFL4.mean, ocular_pressure, MD, and PSD are mainly used for decision (prediction) rules. Most of cases that have RNFL4.mean < = 89.34 are the glaucoma group.

**Table 7:** Usage of features in the decision tree. RNFL4.mean, ocular_pressure, MD, and PSD are mainly used for decision (prediction) rules. Most of cases that have RNFL4.mean < = 89.34 are glaucoma group.

Feature	Usage rate (%)
RNFL4.mean	100.00
ocular_pressure	55.31
PSD	53.75
MD	53.44
age	29.38
cornea_thickness	14.69
GHT	4.06

The rule in line 2 on Fig 5 means that if a case has RNFL4.mean < = 89.33334 and MD < = –4.02 then the case is classified into 1 (glaucoma). The number 126 refers to correctly classified cases in the training dataset by this rule. The rule in line 4 says that if a case has RNFL4.mean < = 89.33334, MD > –4.02, and age > 77, it will be classified into class 0 health control). The number 1 in line 12 means number of misclassified cases.

## Discussion

Glaucoma is a serious disease that can cause complete, permanent blindness, and its early diagnosis is very difficult. In recent years, computer-aided screening and diagnosis of glaucoma has made considerable progress. The accuracy of the prediction model developed in this study was investigated. A visual field index (VFI) value of 97 or higher was defined as early glaucoma. Among the 12 cases of early glaucoma, 11 cases were diagnosed as glaucoma and 1 case was misdiagnosed as normal. Therefore, the diagnosis rate of early glaucoma was 91.7%. One case that was misdiagnosed is discussed below.

**Fig 6:**
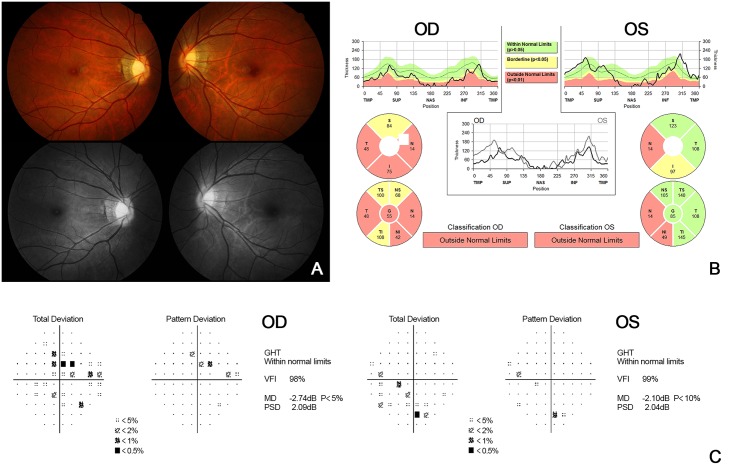
Case 6, color-fundus and red-free fundus photography (A), peripapillary RNFL thickness measured by SD-OCT (B), and automated 30–2 visual field test (C). The presence of a tigroid fundus and peripapillary atrophy was observed, and there was a decrease in the RNFL thickness on the peripapillary RNFL thickness scan. In the visual field test, the abnormalities were judged to be of no clinical significance.

**Fig 7:**
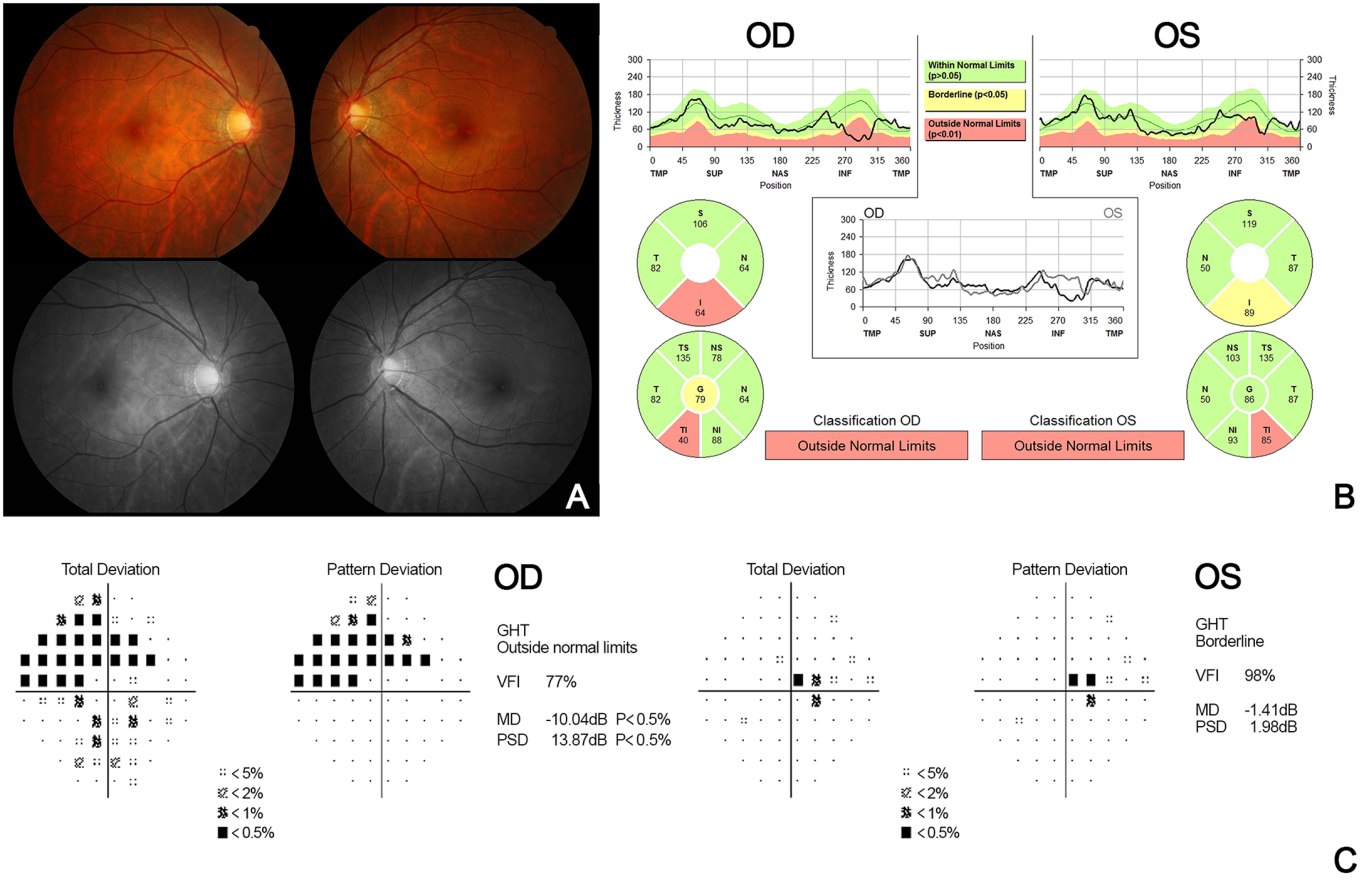
Case 81, color-fundus and red-free fundus photography (A), peripapillary RNFL thickness measured by SD-OCT (B), and automated 30–2 visual field test (C). Fundus photographs show an increased cup-to-disc ratio and RNFL defects in the both eyes. SD-OCT shows decrease in peripapillary thickness of inferotemporal quadrant for both eyes. Visual field defects are apparent in both eyes.

**Fig 8:**
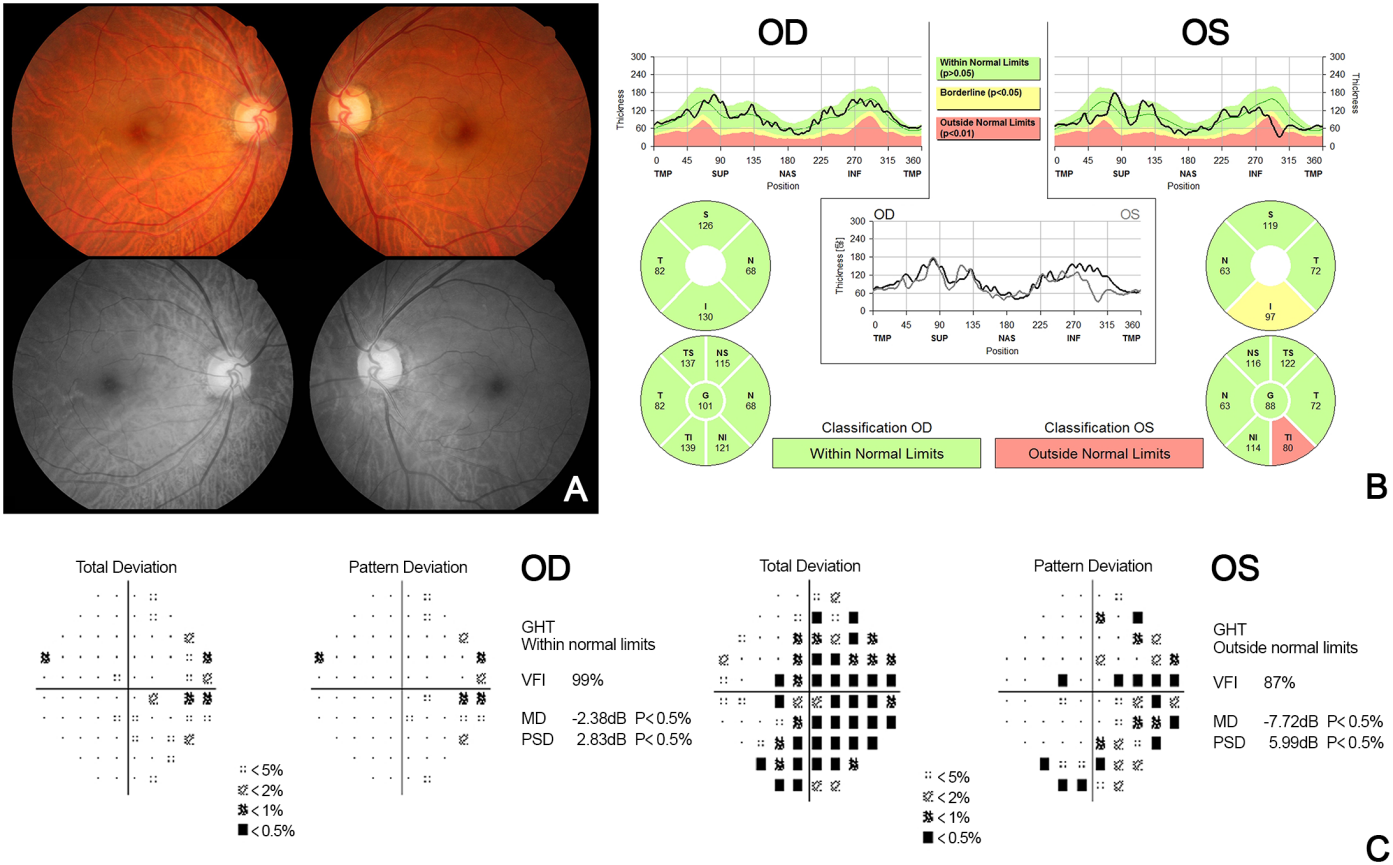
Case 161, color-fundus and red-free fundus photography (A), peripapillary RNFL thickness measured by SD-OCT (B), and automated 30–2 visual field test (C). Fundus photographs show an increased cup-to-disc ratio in both eyes and a RNFL defect in the left eye. SD-OCT shows a decrease in the peripapillary thickness of the infratemporal quadrant of the left eye. The visual field test demonstrates field defect in the left eye.

We reviewed several cases in which there are differences in the results between the clinical diagnosis and the algorithm (C5.0) in detail (Table 8). Firstly, in case 6 (Fig 6), the presence of tigroid fundus and peripapillary atrophy was observed, and there was a decrease in RNFL thickness on the peripapillary RNFL OCT scan. Both eyes were clinically diagnosed as normal by the clinical findings that comprehensively judged the appearance of the optic disc, visual field examination, and normal range of IOP. On the contrary, based on the decision tree generated by the C5.0 algorithm, the mean deviation was reduced to –1.82 dB in both eyes and finally she was diagnosed as having bilateral glaucoma. In this case, the algorithm seems to be diagnosed as glaucoma from the beginning due to the decrease in peripapillary RNFL thickness by high myopia (actually, she had myopia of 6 diopters). Likewise, it is difficult to clinically differentiate between normal and glaucoma because RNFL thickness is often reduced even without glaucoma in patients with high myopia [18–20]. Recently, reports on various OCT parameters and the optic disc morphology for distinguishing normal from glaucoma in high myopia have been published. In the future, it might be possible to improve the accuracy of the algorithm by adding the refraction of the eye and OCT indices, such as macular ganglion cell-inner plexiform layer (GCIPL) thickness, quadrant or clock-hour thickness of RNFL [21–23]. Secondly, in case 81 (Fig 7), clinically, glaucoma was diagnosed in the left eye, but the C5.0 algorithm judged it to be normal. In this patient, both MD and PSD were not significantly worse than the algorithm’s criteria because of the early glaucoma in the left eye. As in this case, we thought that the algorithm had a limitation on the diagnosis of “early glaucoma” with a lack of data in this study. However, this limitation might be improved by using a quadrant or clock-hour thickness of RNFL instead of mean RNFL thickness or by increasing the number of cases. Finally, in case 161 (Fig 8), the review of the decision tree of the C5.0 algorithm revealed that the central corneal thickness was higher than that of the others, and thus it was determined to be normal rather than glaucoma. This error would improve if the number of cases is increased and the standard of central corneal is changed and if the mean RNFL thickness is changed to another OCT index.

**Table 8:** Demographic and clinical data of cases with differences between clinical diagnosis and algorithmic judgment.

Case	Sex/age	Eye	IOP	Central corneal thickness	MD	PSD	Mean RNFL	Clinical diagnosis	C5.0	RF	SVM
6	F/38	OD	17	557	-2.74	2.09	55	H	G	H	G
		OS	18	569	-2.1	2.04	85	H	G	H	G
81	F/35	OD	14	523	-10.04	13.87	79	G	G	G	G
		OS	12	523	-1.41	1.98	86	G	H	H	H
161	F/73	OD	16	573	-2.38	2.83	101	H	H	H	H
		OS	16	589	-7.72	5.99	88	G	H	G	H

(H: Healthy, G: Glaucoma)

Recently, two new machine-learning genome analysis methods, Pse-Analysis (http://bioinformatics.hitsz.edu.cn/Pse-Analysis/) and Pse-in-One [24], have been introduced. These methods support sample feature extraction, optimal parameter selection, model training, cross-validation, and prediction quality evaluation. The methods are optimized for DNA/RNA and protein/peptide sequence data. The in-built support for feature extraction and optimal parameter selection can render these methods invaluable for the diagnosis of Glaucoma. Further investigation and research needs to be conducted to establish if this is a plausible solution for diagnosis of Glaucoma.

Most classification test report results demonstrate that learning models such as RF and SVM deliver a better performance than KNN. Table 4 shows that the accuracy of KNN is similar to other advanced learning models, which means that the derived dataset used in our research has the high quality required for classification but does not clearly reveal the relative performance of the learning models. This establishes that the performance of KNN is expected to deteriorate with an increase in the volume of validation data.

As we mentioned earlier, FN is a more serious error than FP in medical applications. Many learning models support control of the FN rate. In the C5.0 model, we can assign higher error cost to FN than FP. In the RF model, we can modify cutoff values for classes. For example, if we assign stricter values for decisions of healthy control, then we can reduce the number of glaucoma cases that are classified into the healthy control group.

If we want to improve classification accuracy, we can use ensemble learning [25]. It uses multiple learning algorithms to obtain better accuracy. In our cases, we can consider prediction results from the four learning models, and take the majority of results as a final decision.

In our study, we confirm that the machine learning model has many clinical applications and is useful for diagnosing glaucoma. If we gather additional clinical data, we can construct a more accurate, elaborate learning model. In our future studies, we will clarify the cases that on the border between healthy controls and glaucoma cases. For this purpose, we will analyze clinical image data and merge the data with our model. We will also develop diagnostic support software using pre-constructed learning models. With precision medicine gaining considerable attention, we plan to construct new machine-learning models for major ophthalmological diseases and their treatments using precision medicines.
